# The Nutrient Response Transcriptional Regulome of *Arabidopsis*

**DOI:** 10.1016/j.isci.2019.07.045

**Published:** 2019-08-01

**Authors:** Tzvetina Brumbarova, Rumen Ivanov

**Affiliations:** 1 Institute of Botany, Heinrich-Heine University, Universitätstrasse 1, 40225 Düsseldorf, Germany

**Keywords:** Biological Sciences, Omics, Plant Biology, Plant Nutrition, Transcriptomics

## Abstract

Plants respond actively to changes in their environment. Variations in nutrient availability elicit substantial transcriptional reprogramming, and we aimed to systematically describe these adjustments and identify the regulators responsible. Using gene coexpression analysis based on 13 different nutrient availability anomalies, we defined and analyzed nutrient stress response signatures. We identified known regulators and could predict functions in nutrient responses for transcriptional regulators previously associated with other processes, thus linking development and environmental interaction. Three of the identified transcriptional regulators, PIF4, HY5, and NF-Y, known from their role in light signaling, targeted a substantial part of the network and may participate in remodeling the global *Arabidopsis* transcriptome in response to variations of nutrient availability. We present gene coexpression and transcriptional regulation networks, which can be used as tools to further explore regulatory events and dependencies even by users with basic informatics skills.

## Highlights

• Gene coexpression analysis is a powerful tool for elucidating nutrient stress
• Nutrient stress elicits unique signatures of modular transcriptional response
• Master transcriptional regulators coordinate plant growth and nutrient utilization
• Analysis suggests PIF4, HY5, and the NF-Y to be master regulators

## Graphical Abstract

**Figure:**
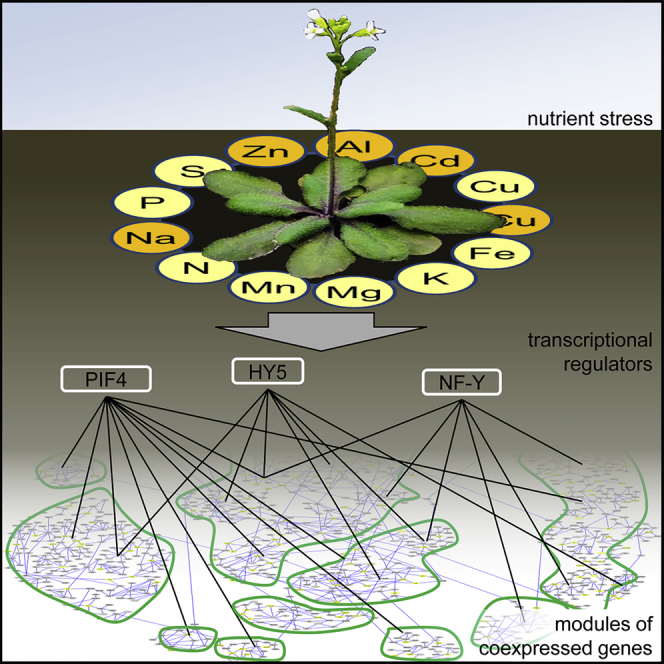

## Introduction

Plants are sessile organisms, and when faced with environmental changes, they have to adapt to them, rather than escape. One challenge for plant survival is the variation in the availability of key nutrients due to daily or seasonal fluctuations in temperature, soil moisture, or pH, as well as the growth of the roots through different soil horizons. Plants respond to changes in nutrient availability by remodeling their transcriptional program. Challenges, such as iron (Fe), copper (Cu), or phosphate (Pi) deficiency, for example, cause the upregulation of specific genes responsible for the high-affinity uptake of these nutrients (Briat et al., 2015, Brumbarova et al., 2015, Penarrubia et al., 2015). However, mineral availability and uptake are interconnected. Changes in factors, such as soil pH, might affect plant's access to more than one nutrient, whereas on the other hand, certain transporters may use more than one nutrient as substrate (Cailliatte et al., 2010, Dubeaux et al., 2018, Korshunova et al., 1999). Thus the cost of boosting uptake of one nutrient might be the overaccumulation of another, interfering with responses and necessitating the activation of additional genes for redistribution and detoxification. Excess of zinc (Zn), for example, mimics Fe deficiency by causing the upregulation of the core Fe uptake genes (Leskova et al., 2017, van de Mortel et al., 2006). Thus, instead of resulting in the differential expression of few key genes, changes in nutrient availability elicit complex transcriptional signatures, often revealing interconnections between nutrient stress responses.

Groups of genes may share similar or close regulation across developmental stages and in response to different stimuli. In gene coexpression analyses, such coregulated genes tend to cluster together in modules. Coexpression analysis has emerged as a powerful tool with predictive value, as the products of coexpressed genes are likely to participate in similar regulatory events, responses to external stimuli, biochemical pathways, etc. (Ivanov et al., 2012, Rodriguez-Celma et al., 2013, Wisecaver et al., 2017).

We have previously used a marker gene-based approach to analyze coexpression under Fe deficiency. For this, we selected genes, which had been consistently regulated under Fe deficiency across a variety of studies, organs, and developmental stages. These were used to generate an Fe-deficiency-oriented gene coexpression network and revealed both expected close coregulation events, such as that between Fe uptake and Fe redistribution-related genes, and significant interplay between the regulation of Fe and other nutrients, such as sulfur (S) (Ivanov and Bauer, 2017, Ivanov et al., 2012). Indeed, an extensive transcriptomic-metabolomic analysis has demonstrated the strong interdependence between the response to low S and low Fe (Forieri et al., 2017). In addition, unexpected interactions could be observed, including a link between Fe and the regulation of the cell cycle and DNA repair in leaves (Ivanov et al., 2012). Thus, gene coexpression analysis is a valuable tool for understanding the coordinated regulation of plant responses to environment.

This led us to map the *Arabidopsis* nutrient response gene coexpression with two fundamental goals in mind. The first goal was to understand to what extent the patterns of coregulated genes within a nutrient response signature are unique to that nutrient and to what extent they are shared with other nutrient regulons. The second was to identify transcriptional regulators relevant for nutrient homeostasis, which might have remained “hidden” due to their strong involvement in basic developmental processes. Therefore, we defined marker genes for 13 different responses to nutrient availability variations and used them to construct a nutrient response gene coexpression network, containing the genes most tightly coexpressed with the markers. Analysis of the network revealed that gene coexpression modules related to fundamental processes, such as cell division, photosynthesis, or hormone signaling, respond to many nutrient conditions. On the other hand, modules containing factors involved in a narrow range of specialized cellular events respond to a limited number of conditions, representing a specific nutrient response. The collective behavior of these modules results in a unique signature characterizing the response of *Arabidopsis* to a particular condition. We generated a list of transcriptional regulators for each module, which revealed sets of specific and general nutrient stress regulators. As a proof of concept, we mapped the gene expression patterns of plants carrying loss- or gain-of-function alleles for several of these regulators to the nutrient response gene coexpression network. We show that at least three of them, PIF4, HY5, and the NF-Y complex, coordinate the responses to nutrient availability with the fundamental developmental program of the plant.

## Results

### Selection of Nutrient Response Marker Genes

To generate the nutrient response gene coexpression network, we first mined available gene expression data from global transcriptomic studies to select marker genes for single stress conditions. Marker genes were selected based on their consistent response to a nutrient stress in independent studies. The selection criteria for each marker gene set are presented in the Transparent Methods. The result of this selection was a list of 408 nutrient response marker genes (Table S1). Among these, 46 genes were shared between more than one stress condition, six were common for at least three conditions, and one, TAIR: At4g19690, was a marker for five of the conditions (Table S2). This is particularly intriguing because At4g19690 encodes the divalent metal transporter IRON-REGULATED TRANSPORTER1 (IRT1) and was previously shown to strongly respond to a large variety of endogenous and external signals (Brumbarova et al., 2015).

**Figure 1:**
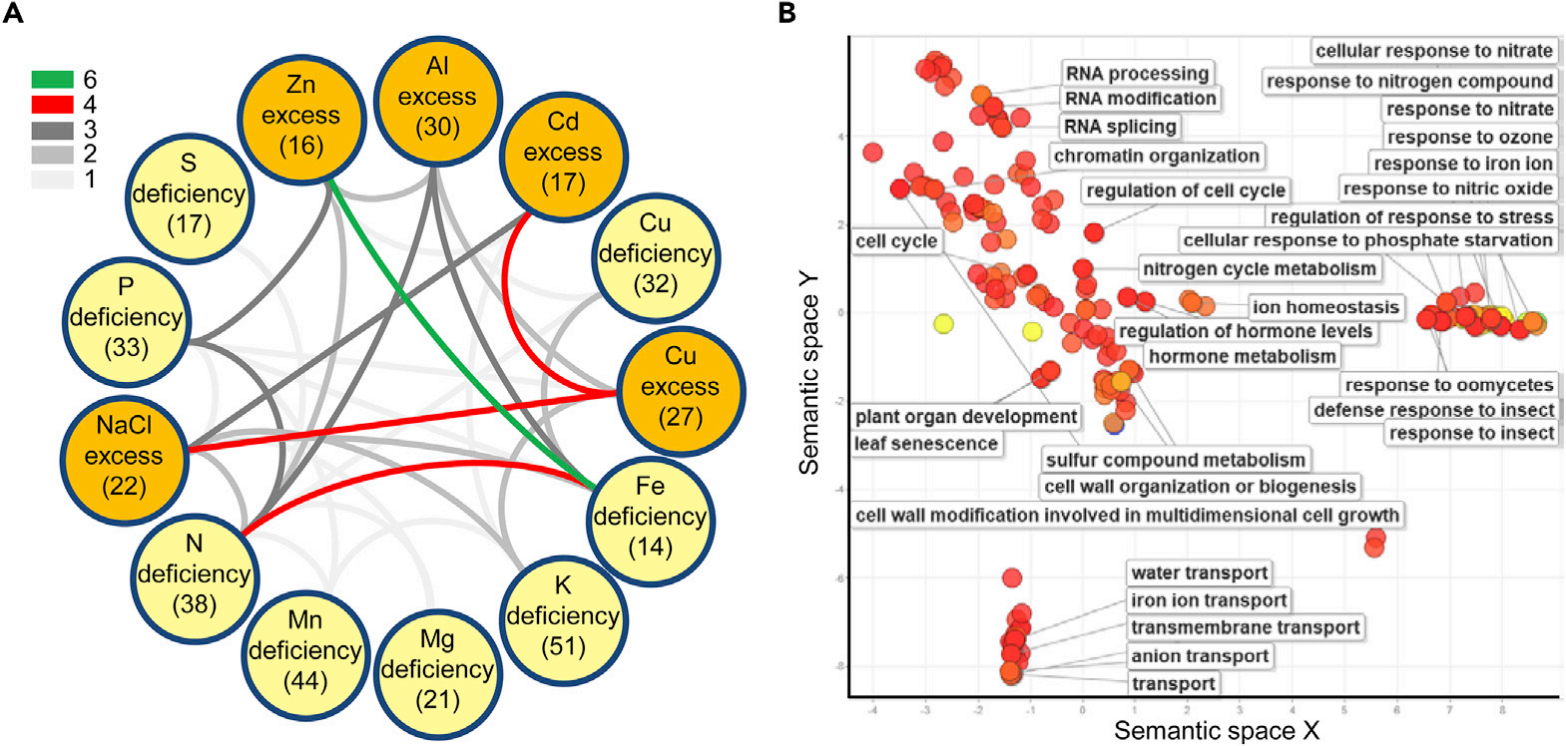
Markers for *Arabidopsis* Nutrient Stress Response (A) Relations between the sets of *Arabidopsis* nutrient stress response marker genes. Circles represent nutrient stresses; deficiencies are in yellow, and excesses in orange. Connections indicate the amount of stress-responsive genes shared between sets. The number of genes unique for each condition is shown in brackets. (B) Semantic similarity scatterplot of GO terms enriched in the 1,647 strongly coexpressed nutrient response genes forming the *Arabidopsis* nutrient response coexpression network.

As over 10% of the stringently selected genes were common for more than one stress response, we investigated the overlap between the marker sets for early indications of similarity in transcriptional responses to different nutrients (Figure 1A). The Cu excess marker set shared four genes with the salt (NaCl) excess and four with the cadmium (Cd) excess set, indicating a common core response mechanism. Similarly, the Fe and nitrogen (N) deficiency sets also shared four genes. As a proof of principle, we observed that our selection algorithm managed to identify the known large overlap in transcriptional response between Fe deficiency and Zn excess, which shared six marker genes (Figure 1A).

### Generation of a Nutrient Response Gene Coexpression Network

We used the 408 marker genes to extract a nutrient response coexpression network using the global *Arabidopsis* gene coexpression set from the ATTED II server (Aoki et al., 2016). This approach was chosen because ATTED II takes into account the transcriptional behavior of genes throughout plant development and in response to a variety of signaling events. Thus the approach provides the necessary resolution to reveal close interconnections between core stress responses and developmental control. To select for the most stable and relevant events, the dataset was stringently trimmed to coexpression strength with a maximum mutual rank value of 5 and to the second-tier neighbor of the query gene. The result of this was a set of 1,647 genes. Gene ontology (GO) and semantic term enrichment analyses of this list revealed categories related to nutrient homeostasis and transport, as well as developmental processes (Tables S3 and S4). Using a semantic similarity-based scatterplot we found that the nutrient homeostasis-related categories grouped closely with pathogen response ones, whereas nutrient and water transport categories formed another distinct group (Figure 1B).

**Figure 2:**
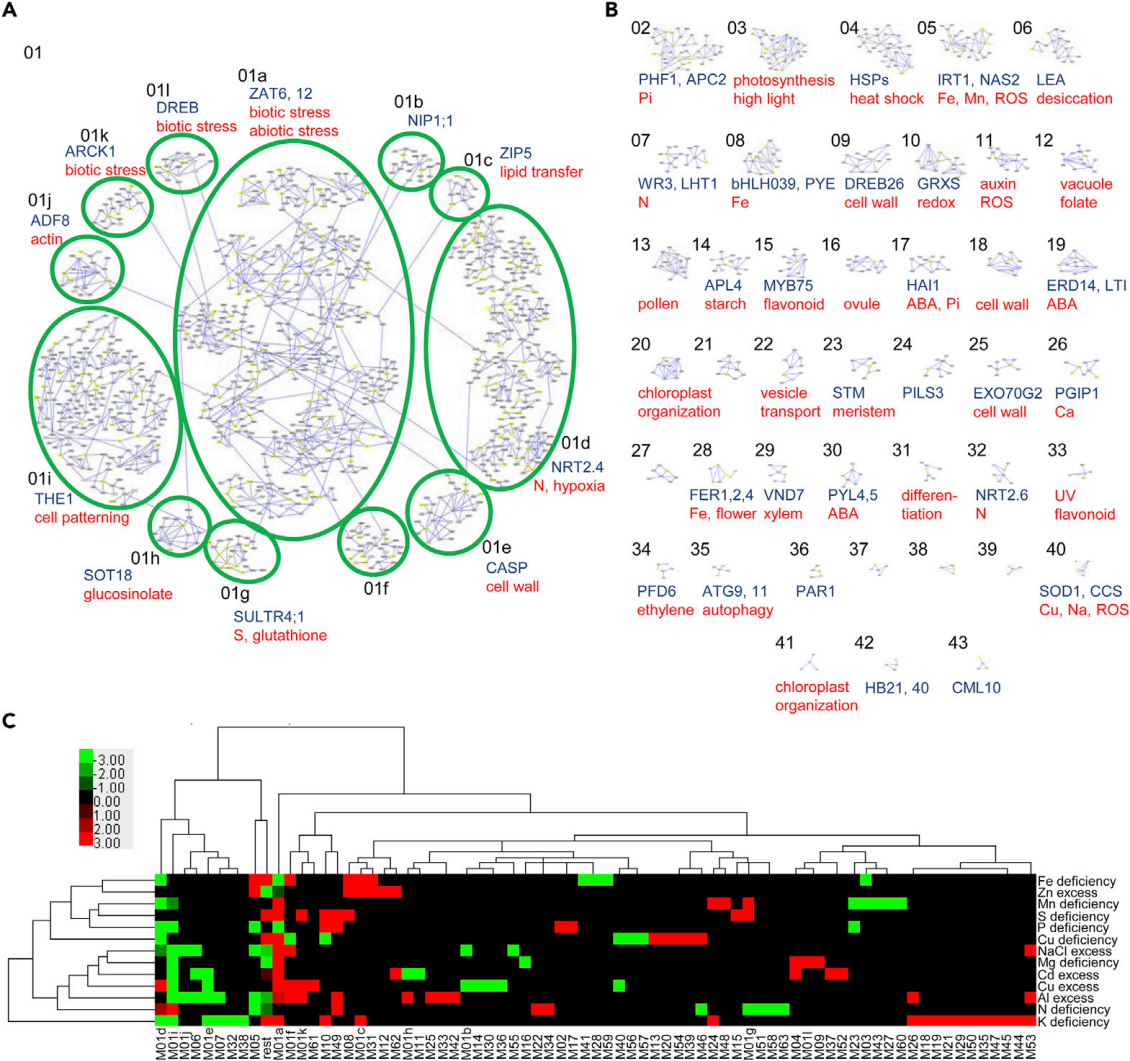
The *Arabidopsis* Nutrient Response Coexpression Network (A and B) Graphical representation of subnetworks 1 (A) and 2–43 (B). In (A), the 12 distinct parts of SN01 are indicated with green ellipses. Where available, example signature proteins encoded within the network (blue) and representative enriched GO/semantic terms categories (red) are shown. (C) Hierarchical clustering of subnetwork performance versus stress response. Green represents subnetwork downregulation, red represents subnetwork upregulation.

The 1,647 nodes (genes) in the coexpression network were connected by 2,107 edges (connections between two strongly coexpressed genes) (Figure 2A, Table S5, Data S1); 1,073 genes, representing more than 65% of the genes in the network, were present in a single group, which we named module 01 (M 01). Based on network connectivity, within M 01 we could identify a total of 12 groups (a-l) of strongly coexpressed genes; 484 genes (29.4%) were present in additional 42 modules containing at least four genes. The remaining 90 genes were in modules of three, two, or as single genes (Figure 2B and Table S5, Data S1). GO and semantic term enrichment analyses showed that many of the modules were associated with nutrient response and homeostasis, as well as response to stress-related hormones, like abscisic acid (ABA), and reactive oxygen species (ROS) metabolism (Figures 2A and 2B, Tables S6 and S7). Other modules, such as M 01i and M 01e were associated with developmental processes, including organ patterning and cell wall modifications, suggesting that the organ growth changes in response to nutrient stress are primed as soon as the physiological response starts (Figures 2A and 2B).

### Analysis of the Network Performance under Nutrient Stress

To understand the performance of each module to the stress conditions analyzed, we assigned a status of upregulated, downregulated, or unchanged, based on the marker genes. For example, M 03 contains six marker genes downregulated and none upregulated under Mn deficiency and was labeled as downregulated (Figure 2C and Table S5). No regulation was assigned when either no marker genes were present for a given condition or in case of ambiguity (a difference of less than 50% between up- or downregulated genes). Ambiguity cases were rare, only 6 of 962, illustrating the robustness of the data.

We performed clustering of the modules according to their response (Figure 2C). The conditions grouped in two major clades, with one containing most of the nutrient excess conditions, together with magnesium (Mg), N, and potassium (K) deficiencies. The second clade contained all micronutrient deficiencies, as well as S and Pi deficiency. An expected exception here was the presence of Zn excess, as responses to Fe deficiency and Zn excess show significant overlap. Modules also grouped in two clades, a smaller one containing rather downregulated modules and a larger one with more diverse response. For this analysis, genes without close coexpression neighbors were grouped together and surprisingly showed very concerted responses to different stresses (Figure 2C rest). This group, as well as M 01a, M 01d, M 01i, and M 05, responded to at least 6 of the 13 conditions. For four of these modules the response dynamics could be attributed to the large amount of marker genes, whereas in M 05 this was due to the diverse responses of the genes in the module. GO analysis of M 05 revealed a biological function related to Fe homeostasis as well as ROS signaling, consistent with the module's performance in our analysis and with published data (Le et al., 2016).

As a proof of the utility of this approach in retrieving the core response genes for single nutrient stresses, we were able to identify several central coexpression modules. M 01g was previously shown to be the core S response cluster (Kopriva et al., 2015), consistent with its GO association and its upregulation upon S deficiency in our analysis (Figures 2A and 2C). The same was true for the three previously described Fe-related modules, M 05, M 08, and M 28 (Ivanov et al., 2012) (Figures 2B and 2C).

Using these criteria, we could identify core response modules for Pi response, which included M 02 and M 17, as upregulated under Pi starvation. M 17 also shows a close coexpression of Pi and ABA response-related genes (Figures 2B and 2C). Among the Pi-downregulated modules were M 01i, already identified as such in the study of Lin et al. (2011), and M 05, known as the central Fe deficiency response regulon (Ivanov et al., 2012) (Figure 2). The latter underlines the reported antagonistic interactions between the responses to Fe and Pi at phenotypic and molecular levels (Gruber et al., 2013, Muller et al., 2015).

Interestingly, both excess and deficiency of Cu caused the downregulation of the general stress response module M 01a and of M 40, containing genes encoding Cu/Zn superoxide dismutases (Figure 2C). At the same time, M 01d and M 01f were both downregulated under Cu deficiency and upregulated under Cu excess. Although these two are among the most nutrient stress-responsive modules (Figure 2C), they may contain core Cu response genes.

In summary, the network describes single nutrient response signatures and allows identification of specific and general nutrient response modules. Furthermore, the network allows to pinpoint modules containing genes with a central function for the response to a specific nutrient.

### Identification of Transcriptional Regulators Targeting the Nutrient Response Network

Transcription factors (TFs) that directly affect responses to nutrient availability are likely to be regulated by stress and are often found coexpressed with their targets as in the case of the Fe homeostasis basic-helix-loop-helix (bHLH) TFs bHLH039 and PYE (Ivanov et al., 2012, Long et al., 2010). On the other hand, the TFs acting upstream in the regulatory networks are potentially less responsive to individual stress conditions (Li et al., 2016) and as they might have additional functions in the plant, their involvement as regulators of nutrient stress response might remain masked. To uncover regulators of nutrient stress responses, each of the modules containing four or more genes was analyzed for being a target of regulatory TFs. We used the Plant Transcription Factor Database v4.0 (http://plantregmap.cbi.pku.edu.cn/network.php) (Jin et al., 2017) and posed three selection criteria (Table S8). The first criterion was literature-reported direct binding and regulation of target promoters by TFs. The second was known binding to target promoter based on chromatin immunoprecipitation-DNA sequencing (ChIP-seq) experiments, because they indicate binding in a cellular context. Third, as not all TFs have been experimentally analyzed and to reduce bias, we also selected TFs showing extremely high score in binding site prediction within the module as a whole. A total of 347 regulators were identified (Table S9), 150 of these (30.7%) for at least two modules (Figure S1A). Thirteen were shown to regulate more than 10% of the analyzed modules (six or more). Four TFs, PHYTOCHROME INTERACTING FACTOR 4 (PIF4, TAIR: At4g43010), LEAFY (LFY, TAIR: At5g61850), ABSCISIC ACID RESPONSIVE ELEMENT-BINDING FACTOR 1 (ABF1, TAIR: At1g49720), and NUCLEAR FACTOR Y, SUBUNIT B2 (NF-YB2, TAIR: At5g47640), regulate more than 10 modules. Forty-seven TFs (13.5%) were already part of the network. More than a quarter of these (13 genes, 27.7%) were among the initially identified nutrient response marker genes. However, none of these marker genes had more than four target modules and, therefore, represented specialized rather than global nutrient response regulators (Figure 1A).

GO (Table S10, Figure 1B) and semantic term enrichment (Table S11) analyses of the transcriptional regulator list showed enrichment of categories such as response to abiotic stimulus and N metabolism on one hand, and development and pattern specification on the other. This shows that nutrient stress responses might be orchestrated already at the level of the basic developmental program of the plant.

The TFs belonged to 34 different protein families. Among the most abundant were MYB with 45 members, NAC (35 members), bZIP (34 members), bHLH (33 members), and WRKY with 30 members (Figure 1C). Considering the size of these families, the MYB, NAC, bZIP, and WRKY TFs were better represented in the set, compared with members of the bHLH and, notably, MADS families. Among the smaller families, HSE and EIL were also well represented, whereas many others were not identified as regulators. This shows that the selection criteria resulted in a non-random set of transcriptional regulators for the nutrient response network.

### Transcriptional Regulation of *Arabidopsis* Nutrient Responses

**Figure 3:**
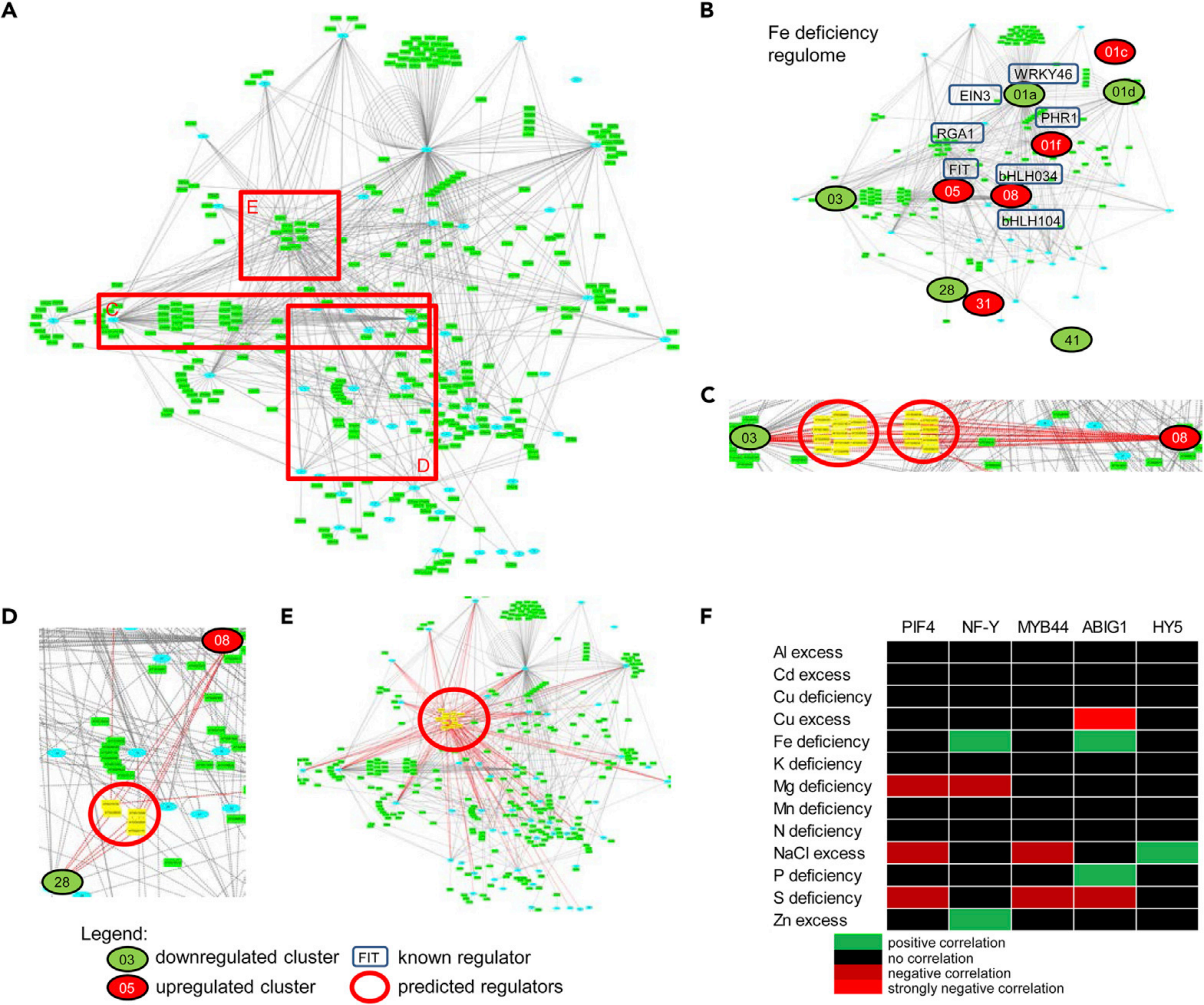
Regulatory Events Governing the Nutrient Response Coexpression Network (A) Overview of the inferred transcriptional control of the nutrient response coexpression network. Blue ellipses represent the subnetworks of the nutrient response coexpression network. Each light green ellipse represents a transcriptional regulator. Directional line indicates a regulatory event. If the transcriptional regulator is a member of a stress response subnetwork, the regulation is represented by a directional line from this subnetwork to the target one. Cases of regulation within the same subnetwork are depicted with curved lines pointing back to the same subnetwork. (B) Example of the recovery of known regulatory events within the iron deficiency regulome. Iron deficiency-responsive subnetworks (dark green for downregulated, red for upregulated) were singled out, together with their associated regulators and target subnetworks. Seven transcription factors with well-documented involvement in gene regulation under iron deficiency could be recovered. (C and D) Multiple common transcriptional regulators for iron-responsive subnetworks 02 (photosynthesis) and 08 (iron homeostasis, C), and 08 and 28 (iron chelation, D). Note that in both cases the regulation of the subnetworks in the couple is opposite. (E) A group of transcription factors predicted to regulate a major portion of the nutrient response coexpression network. (F) Correlation plot for the influence of single transcriptional regulators and nutrient availability on the response of the nutrient response coexpression network. Green cells indicate positive, and red ones, negative correlation.

With the aim of visualizing the potential regulatory relationships, the 347 TFs were mapped to their target modules. The modules were collapsed to highlight the connections between them and the regulators (Figure 3A, Data S2). As a proof of principle, we isolated the modules responding to Fe deficiency together with their regulators. In the regulators list (Table S8), we could identify the central Fe uptake TF FER-LIKE IRON DEFICIENCY INDUCED TRANSCRIPTION FACTOR (FIT) linked to the Fe uptake module (M 05). The approach also correctly identified bHLH034, bHLH104, and WRKY46, as well as the hormone response regulators EIN3 and RGA1, which were all reported to modulate Fe deficiency response (Li et al., 2016, Liang et al., 2017, Lingam et al., 2011, Wild et al., 2016, Yan et al., 2016, Zhang et al., 2015) (Figure 3B). Exploring the network, we could identify additional predicted regulators. Of special interest was a set of 20 TFs, over 50% of which belong to the bZIP family, connecting modules M 03 (photosynthesis) and M 08 (Fe homeostasis) (Figure 3C). Twelve of the TFs (60%) were found to be exclusively targeting these two modules. Accordingly, both processes are strongly affected under Fe deficiency, and genes in both modules are regulated in response to it (Figure 2C).

Similarly, M 08 was well connected with M 28 (genes encoding the Fe storage proteins FERRITINs) (Figure 3D). The common regulators of this module pair include the FHY3-FAR1 couple, involved in far-red response and ABA signaling, and the Pi and Fe deficiency response regulators PHR1 (Liu et al., 2017, Siddiqui et al., 2016). Thus the approach has a high potential of identifying meaningful transcriptional regulators of *Arabidopsis* nutrient responses. Exploring the network further reveals additional potential regulators such as the PHR1 homolog PHL1 and the HRS1 homologs HHO3 and HHO4 as regulators under Pi deficiency (Table S8, Data S2).

As noted above, a small set of regulators appeared to influence a significant number of modules. These general regulators included known developmental regulators, such as LFY; light response proteins, such as PIF4 and ELONGATED HYPOCOTYL5 (HY5); and general transcriptional regulators, such as NF-YB2, all targeting more than six modules (more than 10% of the modules) (Figure 3E and Tables S8 and S9). Many of these regulators are involved in ABA responses and act in a strictly regulated dynamic manner to ensure robust response (Song et al., 2016). In agreement with this, GO and semantic term enrichment analyses showed enrichment of terms like ABA signaling, red-far red response, and transcriptional regulation (Tables S10 and S11). We tested how these regulators influence the *Arabidopsis* nutrient response network. We analyzed transcriptomic data for genes regulated by HY5, MYB44, NF-YB2, PIF4, and ABA INSENSITIVE GROWTH 1 (ABIG1, also known as HAT22) and mapped up- and downregulation events to the network. To estimate the role of NF-YB2, we used an NF-YA transcriptome analysis as these two function in a heterotrimeric complex, which includes a third subunit, NF-YC (Siefers et al., 2009). The significant regulatory events were mapped to our nutrient response network (Table S12) to define how each of the TFs influences each module. We then evaluated how many of the differentially regulated modules were initially predicted to be targets of the TF. For three of the regulators, HY5, NF-Y, and PIF4, we found a very good correlation between prediction and experimental data (Table S13). In all three cases, the overlap exceeded 50%, with the NF-Y complex reaching 85%. For MYB44 and ABIG1, the overlap was much lower; however, this might also reflect the small amount of differentially expressed genes identified in these studies (totally 119 for MYB44 and 40 for ABIG1, compared with a minimum of 290 for other three TFs). Thus, HY5, PIF 4, and the NF-Y complex might be capable of influencing large subsets of plants' responses to nutrient availability and might therefore be considered global nutrient response regulators.

To estimate whether any of the regulators has a major role in defining the stress response transcriptome signature, we performed pairwise comparisons between the regulomes of the TFs and the nutrient stress conditions. We ranked the pairs according to their Pearson product-moment correlation coefficient, where positive correlation suggested a significant contribution of the TF to promoting the stress-related expression signature. Negative correlation suggested that the TF contributes to the maintenance of modules that do not respond to the particular stress or that it does not participate in these responses. Values between −0.24 and 0.24 were not considered in the analysis. The data showed that the NF-Y complex and HY5 significantly contribute to the Fe deficiency/Zn excess and salt stress transcriptome, respectively. ABIG1 transcriptome correlated with several stress conditions, positively with Fe and P deficiencies, negatively with S deficiency, and strongly negatively with the Cu excess transcriptome. Surprisingly, PIF4 transcriptome showed three negative correlations, to the global response to salt excess as well as deficiencies in the macronutrients S and Mg (Figure 3F). Thus, PIF4 may regulate its target modules in a manner opposite to the expected stress response expression signature, suggesting that under these nutrient supplies the activity of PIF4 may need to remain low. The data demonstrate that the full response signature to nutrient stresses requires the concerted interaction of many regulators. At the same time, systems-level analyses allow to propose the contribution of single TFs to plant responses to the environment.

## Discussion

Transcriptional responses to variations in nutrient availability are complex and interconnected. In this study, we identified marker genes for a large number of conditions where physiologically relevant nutrient supply is perturbed. Based on these genes, we generated a nutrient response gene coexpression network for the model plant *Arabidopsis*. With the help of this network, we identified global and stress-specific modules of coexpressed genes and were able to uncover transcriptional regulators potentially associated with nutrient responses.

We show that the transcriptional response of *Arabidopsis* to nutrient stress has a modular structure, based on the up- or downregulation of units of coexpressed genes. Each stress elicits a specific response pattern, which can be defined as a signature. Importantly, certain modules occur often within the here investigated signatures and therefore likely represent the basic *Arabidopsis* stress response. A prominent example for this is M 01a, which contains a large amount of tightly coexpressed genes and reacts to multiple nutrient conditions. Enrichment of GO categories and semantic terms for responses to biotic and abiotic stress fully supports such a role. Indeed, other studies have identified coexpression modules corresponding to M 01a as central in the responses to abiotic (Ransbotyn et al., 2015) and biotic (Amrine et al., 2015) stress. Despite having a relatively low number of genes, some of the smaller modules, such as M 01f and M 05, also fall in this category. M 05 is intriguing, as it contains the core *Arabidopsis* Fe acquisition genes and is usually associated with Fe deficiency response (Ivanov et al., 2012). Indeed, the module member IRT1 was identified as a marker for the highest number of nutrient responses. IRT1 expression variations have also been observed for a variety of other plant responses (Blum et al., 2014, Brumbarova et al., 2015). On the contrary, the module M 08 involved in Fe homeostasis and redistribution responds specifically to Fe deficiency and Zn excess. These facts about the Fe-related modules highlight two important points. First, that managing the availability of essential but potentially toxic metals, such as Fe, Zn, and Mn, is a critical event in environmental change response. Second, the control of metal availability under suboptimal conditions is mainly achieved at the acquisition stage.

A key contribution of our approach to understanding *Arabidopsis* nutrient responses was the identification of additional potential transcriptional regulators together with the known nutrient-related TFs. Their association with combinations of target gene modules uncovers coexpression relationships within a nutrient response signature. A particularly interesting case is the high degree of coregulation within the module couples M 03-M 08 and M 08-M 28, all regulated by Fe deficiency.

The main GO and semantic term categorization of M 03, photosynthesis, is a process strongly dependent on Fe and severely affected by its absence. Sets of TFs were identified as common regulators for each of the module couples, providing a direct link between different steps of Fe homeostasis and utilization. Many of these TFs, belonging to the bZIP family, might be functionally redundant, explaining the fact that their function in Fe homeostasis has so far remained obscure. Thus, our approach suggests previously unknown Fe homeostasis regulators. It is important to note, however, that in both cases the modules were oppositely regulated under Fe deficiency. This serves as a reminder that TFs do not work alone but in complexes and the combination of partners in the complex might determine its activity and function as an activator or repressor. Currently, coexpression networks do not account for potential complex assembly. However, the fact that modules are often targets of multiple TFs suggests that such information may be inherently present. Another important consideration for network exploration is that the regulated processes occur in complex organs containing many different cell types. Specialized cells in discreet hormone-regulated zones along the root perform different nutrient uptake steps (Blum et al., 2014, Kiba and Krapp, 2016, Perea-Garcia et al., 2013). Lateral distribution of nutrients depends on different developmental and element-specific barriers (Barberon et al., 2016, Claus et al., 2013, Duan et al., 2018, Lin et al., 2009). Owing to the enormous dataset feeding the ATTED-II *Arabidopsis* database, many of these effects are actually taken into account in our coexpression network. For example, the M 01e module, enriched in endodermis-specific CASP genes, reflects the spatially restricted expression pattern of nutrient-responsive genes. Additional examples include, but are not limited to, the cell patterning-related module M 01i and the xylem differentiation-related M 29, reflecting the reprogramming of root development in response to nutrient availability (Gruber et al., 2013).

Among the identified transcriptional regulators we find several well-characterized proteins involved in multiple signaling pathways. Apparently, the role of such TFs in nutrient stress has remained masked, underlining the utility of the gene coexpression approach in drawing attention to unsuspected functions of characterized regulators. A combination of target gene prediction and gene expression analysis of three regulators, HY5, PIF4, and the NF-Y complex, points toward them being potential prominent modulators of nutrient responses. Interestingly, all three have been isolated and studied mainly in connection to plant light responses, suggesting that light plays a key role both as energy and information source in response to environmental changes affecting nutrient availability. Recently, HY5 was demonstrated as a key TF transported from leaves to roots, coupling light perception, root growth, and N homeostasis (Chen et al., 2016). Our analysis has independently identified HY5 as a regulator of not only N responses (M 01g) but also S and Fe homeostasis (modules M 01g and M 08) (Tables S12 and S13). PIF4 is a key signaling crossing point, coordinating plant morphogenesis in response to multiple environmental signals and the circadian clock (Leivar and Monte, 2014, Paik et al., 2017, Quint et al., 2016). Together with HY5, PIF4 is involved in shade avoidance regulation (Gommers and Monte, 2018), which requires an evasive growth response fueled by proper nutrient supply. Fittingly, PIF4 also binds the promoters of biosynthesis and signaling of many auxins (Iglesias et al., 2018, van Gelderen et al., 2018), acting as a connection point between perception of changed growth requirements and growth control. PIF4 was also shown to regulate the response to salt stress by downregulating the transcription of the NAC family TFs JUNGBRUNNEN1 (JUB1) and ORESARA1 (ORE1) (Balazadeh et al., 2010, Wu et al., 2012). Consistently, in our analysis PIF4 and ORE1 are among the regulators of the general stress response module M 01a (part of the salt signature) and M 01l, which is part of the Mg response signature, containing seven AP2/EREBP (APETALA2/ETHYLENE-RESPONSIVE ELEMENT BINDING PROTEINS) TFs. NF-Y is a heterotrimeric complex known to participate in a multitude of environmental responses, including salt stress (Li et al., 2013). The complex consists of three subunits, A, B, and C, each of which is encoded by several genes, allowing formation of multiple complex versions. Here we identified the B2 subunit as a factor targeting at least 12 of the coexpression modules. In addition, we found NF-YC2 to target two of the modules—M 02, involved in Pi homeostasis and upregulated under Pi starvation, and M 14 participating in starch biosynthesis, part of the Cu excess signature. Both of these modules are also NF-YB2 targets, and their response was shown experimentally to be NF-Y dependent. Thus the analysis suggests additional functions for these three factors as global regulators of plant nutrient stress response.

In summary, the *Arabidopsis* nutrient response gene coexpression network offers an understandable and explorable network, which can be used by biologists without advanced informatics skills as a powerful information source on transcriptional regulation of plant nutrient stress responses.

### Limitations of the Study

We would like to note that the identification of transcriptional regulators for the *Arabidopsis* nutrient response gene coexpression network was in part based on ChIP-seq data. Owing to the fact that only a subset of all transcriptional regulators have been analyzed by this method, one has to keep in mind that the end result of the analysis might be slightly biased toward these regulators. The lists have been balanced by adding published and predicted regulators; however, one should keep in mind that some key regulators might have been missed and may be identified should the analysis be repeated in the future.

In addition, we have demonstrated that the approach can be used for uncovering potential regulators. However, we need to stress that the obtained results will represent predictions and it is of extreme importance that identified regulators are experimentally validated in the respective system before proceeding with further research questions and hypotheses.

## Methods

All methods can be found in the accompanying Transparent Methods supplemental file.

## Supplemental Information

Document S1. Transparent Methods and Figure S1

Document S2. Tables S1–S13

Data S1. The *Arabidopsis* Nutrient Response Gene Coexpression Network (Cytoscape Source File), Related to Figure 2

Data S2. Transcriptional Regulation of the *Arabidopsis* Nutrient Response Gene Coexpression Network (Cytoscape Source File), Related to Figure 3
