# Supplementary material for: The Nutrient Response Transcriptional Regulome of Arabidopsis
Source: iScience. 2019 Aug 1;19:358–68. doi: 10.1016/j.isci.2019.07.045 (PMC6702435; doi:10.1016/j.isci.2019.07.045)
Supplement: Document S1. Transparent Methods and Figure S1 [file mmc1.pdf]

ISCI, Volume *19*

Supplemental Information

The Nutrient Response

Transcriptional Regulome of *Arabidopsis*

Tzvetina Brumbarova and Rumen Ivanov

Supplemental Figure

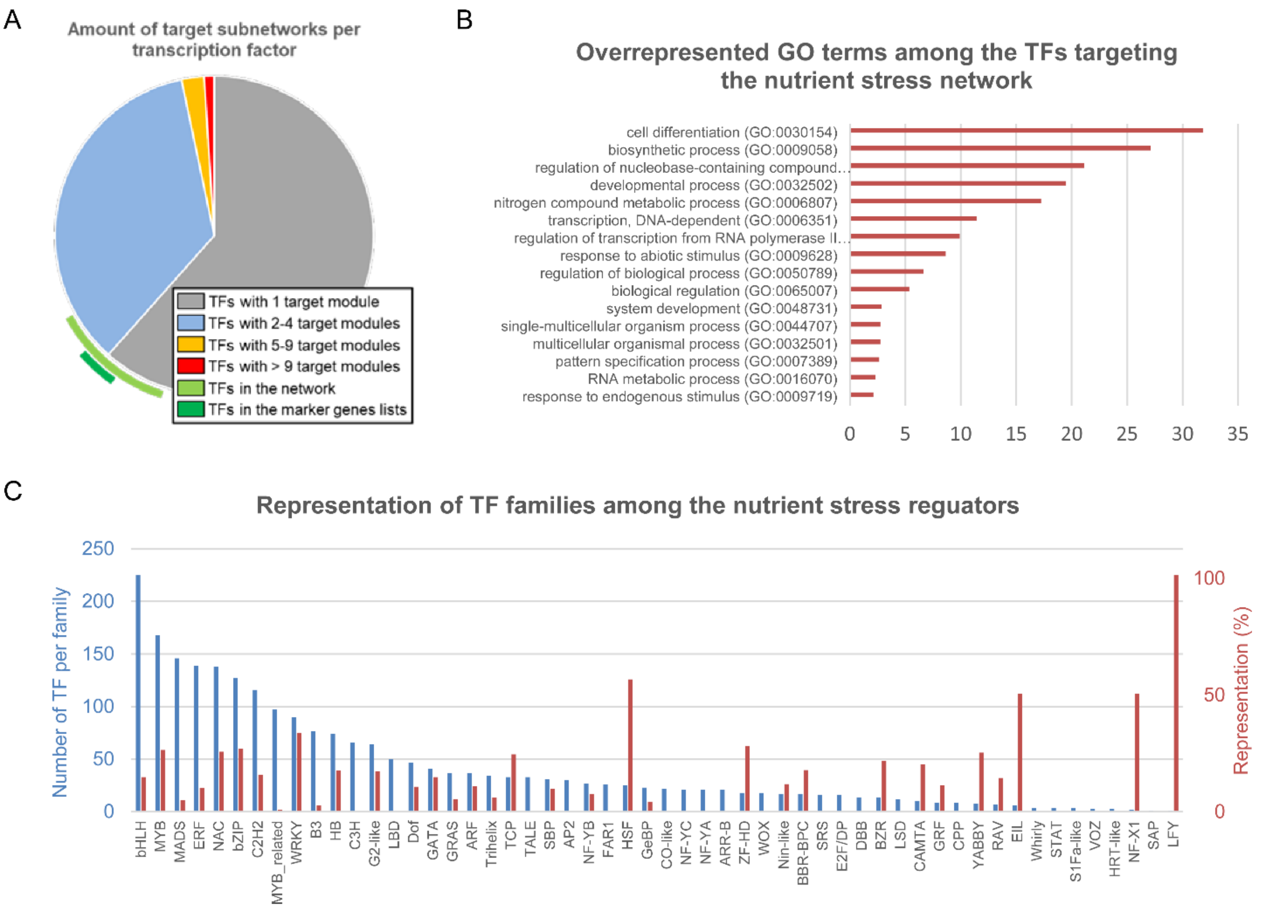

Figure S1. Transcriptional regulators of the nutrient response coexpression network, Related to Figure 3. (A) Amount of subnetworks targeted per transcription factor. (B) List of enriched GO terms for the identified transcriptional regulators. Mainly cell differentiation and development-related categories were enriched in the set. The values represent negative base 10 logarithm of the enrichment P-value. (C) Representation of transcription factor families among the list of nutrient stress transcriptional regulators. The total number of members within the transcription factor family is represented in blue bars. The percentage of proteins from each family represented in the nutrient response regulator list is shown in claret bars.

Transparent Methods

Selection of nutrient stress marker genes

Global transcriptomic studies were mined to select nutrient stress marker genes. Since these studies used large variety of plant genotypes, growth conditions and treatment procedures, we applied two major selection criteria. First, the experiment has to use natural accessions, not loss-of-function or gain-of-function alleles. Second, the experiment should allow the comparison between a control condition and a condition involving altered nutrient supply. The Al toxicity list contains the 15 most significantly upregulated and 15 most significantly downregulated genes from the study Zhao et al. (2009). The lists were complemented with the genes which were identified in both Zhao et al. (2009) and Kumari et al. (2008). The latter study assayed considerably less genes, which is the reason why the list is primarily based on Zhao et al., (2009). The Cd toxicity list was compiled based on the genes considered to be significantly up- or downregulated in the studies of Zhao et al. (2009) and Weber et al. (2006). Cu deficiency response list includes the strongest-reacting genes in roots selected in the study of Bernal et al. (2012). The Cu toxicity list was compiled from the genes considered to be significantly up- or downregulated in the studies of Zhao et al. (2009) and Weber et al. (2006). All downregulated genes identified in both studies were selected. The list of upregulated genes includes the 20 strongest regulated genes identified in both studies. The Fe-deficiency response list consists of the most stably up- and downregulated genes presented in Mai et al. (2015) (see also references therein). One additional gene, At4g19680, was included as it was identified as stable iron-deficiency marker gene in the study of Ivanov et al. (2012). The K deficiency response list was based on the study by Armengaud et al. (2004). Among the genes shown to be up- or downregulated under K starvation, those genes were selected, which showed opposite regulation within two hours to K resupply. The Mg-deficiency list was compiled by comparing the early response genes in the roots identified as consistently regulated in at least two time points in the study of Hermans et al. (2010) and the differentially-regulated genes in root samples in the study of Niu et al. (2016). The comparison yielded 20 upregulated genes, to which the three downregulated in the Hermans et al. (2010) study were added. The Mn-deficiency list contains the genes significantly upregulated in roots in the studies of Yang et al. (2008) and Rodriguez-Celma et al. (2016), whose protein products a consistent Mn-dependent regulation was also

observed in the study of Rodriguez-Celma et al. (2016). The N deficiency response list includes the genes found in the study of Krapp et al. (2011) to react early and steadily to N starvation in the root. The list contains the highest-regulated genes in the categories "Rapidly changed and stayed high/low" and "Differential expression increased during starvation". Na, though considered functional rather than essential nutrient for Arabidopsis, was included due to its strong interaction with the homeostasis of other nutrients, such as K and its toxic effects upon excess. The Na toxicity list was compiled based on the genes considered to be significantly up- or downregulated in the study of Zhao et al., (2009) and genes registered as differentially-regulated in roots in the NaCl time course experiment of Dinneny et al. (2008). The Pi response list was generated based on the early root phosphate deficiency-induced genes identified by Misson et al. (2005). The upregulated genes were compared to the genes found to be upregulated by phosphate deficiency and downregulated within 3 hours of phosphate resupply in the study of Morcuende et al. (2007). The 20 top upregulated genes in the Misson et al., which were also identified in Morcuende et al., together with the 20 top downregulated genes according to Misson et al. were selected for further analysis. The S deficiency list was compiled by comparing the root transcriptome data of Nikiforova et al. (2003), Bielecka et al. (2014) and the selection of most highly expressed genes in the study of Forieri et al. (2013). Genes discovered in at least two of the studies were considered. Only upregulated genes matched the criteria. The Zn toxicity list was compiled from the genes considered to be significantly up- or downregulated in the studies of van de Mortel et al. (2006) and Landa et al. (2015).

Analysis of marker gene overlap between nutrient stress conditions

The list of selected genes, irrespective of the type of regulation, were processed by the online Venn diagram software at http://bioinformatics.psb.ugent.be/webtools/Venn/.

Generation of the gene coexpression network

The 408 selected genes were used as query in the Arabidopsis coexpression database ATTED-II version 9.3 (Aoki et al, 2016). The generated network was loaded in Cytoscape, where only genes very strongly coexpressed with the query were selected for further analysis. The criteria were Mutual rank value of no more than 5 and a limit to the second-level neighbor of the query gene.

Gene ontology and semantic term enrichment analysis

GO terms over and under representation analysis was performed using the Protein Analysis THrough Evolutionary Relationships (PANTHER) classification tool (http://pantherdb.org/) (Mi et al, 2016). Semantic similarity-based scatterplot analysis was performed with the help of REVIGO software (http://revigo.irb.hr) (Supek et al, 2011). For the retrieval of enriched semantic terms in gene descriptions, the GeneCloud tool (www.m2sb.org) (Krouk et al, 2015) was used. Terms occurring in at least 3 genes were taken into consideration. A false discovery rate (FDR) threshold of 0.05 based on the Benjamini–Yekutieli procedure was applied.

Hierarchical clustering of subnetwork transcriptional response to stress

The presence of each of the marker genes within the subnetwork in each of the initial gene lists was checked. Upon presence in the list of upregulated genes under the checked condition, the gene was assigned a value of 1 for that condition. Oppositely, if the gene was present in the list of downregulated genes, it received value of -1. For each condition, a collective value was assigned to the subnetwork, based on the values of its regulated genes under that condition. The values ranged from -50, representing only negatively regulated genes, to 50 representing only positively regulated genes. A value of 0 showed either no or unclear regulation under that condition. The same approach was followed for clustering the transcription factor targets. Gene Cluster 3.0 software (http://bonsai.hgc.jp/~mdehoon/software/cluster/software.htm, C Clustering Library version 1.54) was used for hierarchical clustering of subnetworks. Euclidean distance was used as similarity metric and complete linkage was employed as clustering method. The results were visualized using the Java Tree View software (http://jtreeview.sourceforge.net, version 1.1.6r4).

Identification of potential transcriptional regulators

The discovery of transcription factors known or predicted to be involved in the regulation of the genes in the subnetworks was performed using the Plant Transcription Factor Database v4.0 (http://plantregmap.cbi.pku.edu.cn/network.php) (Jin et al, 2017). Three separate lists were created for each cluster. The first list was prediction-based and contained transcription factors predicted to preferentially bind promoters in each cluster

(http://plantregmap.cbi.pku.edu.cn/tf_enrichment.php). Only transcription factors with enrichment P-value lower than 0.0001 were considered for further analysis. The second list was based on chromatin immunoprecipitation followed by DNA sequencing (ChIP-seq) data for transcription factor binding. Transcription factors shown to bind at least 5 % of the promoters in a given cluster and appeared at least twice per cluster were further considered. The third list contained literature curated regulatory data. Transcription factors that met any of the criteria for a certain cluster were further labeled as regulators of these clusters.

Transcription factor families were assigned with the help of the Arabidopsis transcription factor database (http://agris-knowledgebase.org/AtTFDB/).

Selection of genes influenced by the nutrient response network regulators

The lists of genes significantly differentially regulated by selected regulators of the nutrient response network were taken from the following studies: PIF4 (Gangappa et al, 2017), MYB44 (Jaradat et al, 2013), NF-Y (Leyva-Gonzalez et al, 2012), ABIG1 (Liu et al, 2016), HY5 (Kleine et al, 2007). To achieve uniformity and comparability of the data, genes were assigned to be either upregulated or downregulated by the presence of the transcription factor.

Correlation analysis between nutrient and transcription factor effects on the regulation of the nutrient response coexpression network

The transcriptomic responses of the nutrient coexpression network to variation in nutrients (Table S2) and the effect of transcription factors (Table S8) were compared pairwise to each other. The Pearson Product-Moment Correlation Coefficient was calculated for each pair to allow comparison between the pairs.

Supplemental References

Aoki, Y., Okamura, Y., Tadaka, S., Kinoshita, K. & Obayashi, T. (2016) ATTED-II in 2016: A Plant Coexpression Database Towards Lineage-Specific Coexpression. *Plant Cell Physiol*, 57(1), e5.

Armengaud, P., Breitling, R. & Amtmann, A. (2004) The potassium-dependent transcriptome of Arabidopsis reveals a prominent role of jasmonic acid in nutrient signaling. *Plant Physiol*, 136(1), 2556-76.

Bernal, M., Casero, D., Singh, V., Wilson, G. T., Grande, A., Yang, H., Dodani, S. C., Pellegrini, M., Huijser, P., Connolly, E. L., Merchant, S. S. & Kramer, U. (2012) Transcriptome sequencing identifies SPL7-regulated copper acquisition genes FRO4/FRO5 and the copper dependence of iron homeostasis in Arabidopsis. *Plant Cell*, 24(2), 738-61.

Bielecka, M., Watanabe, M., Morcuende, R., Scheible, W. R., Hawkesford, M. J., Hesse, H. & Hoefgen, R. (2014) Transcriptome and metabolome analysis of plant sulfate starvation and resupply provides novel information on transcriptional regulation of metabolism associated with sulfur, nitrogen and phosphorus nutritional responses in Arabidopsis. *Front Plant Sci*, 5, 805.

Dinneny, J. R., Long, T. A., Wang, J. Y., Jung, J. W., Mace, D., Pointer, S., Barron, C., Brady, S. M., Schiefelbein, J. & Benfey, P. N. (2008) Cell identity mediates the response of Arabidopsis roots to abiotic stress. *Science*, 320(5878), 942-5.

Forieri, I., Wirtz, M. & Hell, R. (2013) Toward new perspectives on the interaction of iron and sulfur metabolism in plants. *Front Plant Sci*, 4, 357.

Gangappa, S. N., Berriri, S. & Kumar, S. V. (2017) PIF4 Coordinates Thermosensory Growth and Immunity in Arabidopsis. *Curr Biol*, 27(2), 243-249.

Hermans, C., Vuylsteke, M., Coppens, F., Craciun, A., Inze, D. & Verbruggen, N. (2010) Early transcriptomic changes induced by magnesium deficiency in Arabidopsis thaliana reveal the alteration of circadian clock gene expression in roots and the triggering of abscisic acid-responsive genes. *New Phytol*, 187(1), 119-31.

Ivanov, R., Brumbarova, T. & Bauer, P. (2012) Fitting into the harsh reality: regulation of iron-deficiency responses in dicotyledonous plants. *Mol Plant*, 5(1), 27-42.

Jaradat, M. R., Feurtado, J. A., Huang, D., Lu, Y. & Cutler, A. J. (2013) Multiple roles of the transcription factor AtMYBR1/AtMYB44 in ABA signaling, stress responses, and leaf senescence. *BMC Plant Biol*, 13, 192.

Jin, J., Tian, F., Yang, D. C., Meng, Y. Q., Kong, L., Luo, J. & Gao, G. (2017) PlantTFDB 4.0: toward a central hub for transcription factors and regulatory interactions in plants. *Nucleic Acids Res*, 45(D1), D1040-D1045.

Kleine, T., Kindgren, P., Benedict, C., Hendrickson, L. & Strand, A. (2007) Genome-wide gene expression analysis reveals a critical role for CRYPTOCHROME1 in the response of Arabidopsis to high irradiance. *Plant Physiol*, 144(3), 1391-406.

Krapp, A., Berthome, R., Orsel, M., Mercey-Boutet, S., Yu, A., Castaings, L., Elftieh, S., Major, H., Renou, J. P. & Daniel-Vedele, F. (2011) Arabidopsis roots and shoots show distinct temporal adaptation patterns toward nitrogen starvation. *Plant Physiol*, 157(3), 1255-82.

Krouk, G., Carre, C., Fizames, C., Gojon, A., Ruffel, S. & Lacombe, B. (2015) GeneCloud Reveals Semantic Enrichment in Lists of Gene Descriptions. *Mol Plant*, 8(6), 971-3.

Kumari, M., Taylor, G. J. & Deyholos, M. K. (2008) Transcriptomic responses to aluminum stress in roots of Arabidopsis thaliana. *Mol Genet Genomics*, 279(4), 339-57.

Landa, P., Prerostova, S., Petrova, S., Knirsch, V., Vankova, R. & Vanek, T. (2015) The Transcriptomic Response of Arabidopsis thaliana to Zinc Oxide: A Comparison of the Impact of Nanoparticle, Bulk, and Ionic Zinc. *Environ Sci Technol*, 49(24), 14537-45.

Leyva-Gonzalez, M. A., Ibarra-Laclette, E., Cruz-Ramirez, A. & Herrera-Estrella, L. (2012) Functional and transcriptome analysis reveals an acclimatization strategy for abiotic stress tolerance mediated by Arabidopsis NF-YA family members. *PLoS One*, 7(10), e48138.
Liu, T., Longhurst, A. D., Talavera-Rauh, F., Hokin, S. A. & Barton, M. K. (2016) The Arabidopsis transcription factor ABIG1 relays ABA signaled growth inhibition and drought induced senescence. *Elife*, 5.
Mai, H. J., Lindermayr, C., von Toerne, C., Fink-Straube, C., Durner, J. & Bauer, P. (2015) Iron and FER-LIKE IRON DEFICIENCY-INDUCED TRANSCRIPTION FACTOR-dependent regulation of proteins and genes in Arabidopsis thaliana roots. *Proteomics*, 15(17), 3030-47.
Mi, H., Poudel, S., Muruganujan, A., Casagrande, J. T. & Thomas, P. D. (2016) PANTHER version 10: expanded protein families and functions, and analysis tools. *Nucleic Acids Res*, 44(D1), D336-42.
Misson, J., Raghothama, K. G., Jain, A., Jouhet, J., Block, M. A., Bligny, R., Ortet, P., Creff, A., Somerville, S., Rolland, N., Doumas, P., Nacry, P., Herrerra-Estrella, L., Nussaume, L. & Thibaud, M. C. (2005) A genome-wide transcriptional analysis using Arabidopsis thaliana Affymetrix gene chips determined plant responses to phosphate deprivation. *Proc Natl Acad Sci U S A*, 102(33), 11934-9.
Morcuende, R., Bari, R., Gibon, Y., Zheng, W., Pant, B. D., Blasing, O., Usadel, B., Czechowski, T., Udvardi, M. K., Stitt, M. & Scheible, W. R. (2007) Genome-wide reprogramming of metabolism and regulatory networks of Arabidopsis in response to phosphorus. *Plant Cell Environ*, 30(1), 85-112.
Nikiforova, V., Freitag, J., Kempa, S., Adamik, M., Hesse, H. & Hoefgen, R. (2003) Transcriptome analysis of sulfur depletion in Arabidopsis thaliana: interlacing of biosynthetic pathways provides response specificity. *Plant J*, 33(4), 633-50.
Niu, Y., Ahammed, G. J., Tang, C., Guo, L. & Yu, J. (2016) Physiological and Transcriptome Responses to Combinations of Elevated CO2 and Magnesium in Arabidopsis thaliana. *PLoS One*, 11(2), e0149301.
Rodriguez-Celma, J., Tsai, Y. H., Wen, T. N., Wu, Y. C., Curie, C. & Schmidt, W. (2016) Systems-wide analysis of manganese deficiency-induced changes in gene activity of Arabidopsis roots. *Sci Rep*, 6, 35846.
Supek, F., Bosnjak, M., Skunca, N. & Smuc, T. (2011) REVIGO summarizes and visualizes long lists of gene ontology terms. *PLoS One*, 6(7), e21800.
van de Mortel, J. E., Almar Villanueva, L., Schat, H., Kwekkeboom, J., Coughlan, S., Moerland, P. D., Ver Loren van Themaat, E., Koornneef, M. & Aarts, M. G. (2006) Large expression differences in genes for iron and zinc homeostasis, stress response, and lignin biosynthesis distinguish roots of *Arabidopsis thaliana* and the related metal hyperaccumulator *Thlaspi caerulescens*. *Plant Physiol*, 142(3), 1127-47.
Weber, M., Trampczynska, A. & Clemens, S. (2006) Comparative transcriptome analysis of toxic metal responses in Arabidopsis thaliana and the Cd(2+)-hypertolerant facultative metallophyte Arabidopsis halleri. *Plant Cell Environ*, 29(5), 950-63.
Yang, T. J., Perry, P. J., Ciani, S., Pandian, S. & Schmidt, W. (2008) Manganese deficiency alters the patterning and development of root hairs in Arabidopsis. *J Exp Bot*, 59(12), 3453-64.
Zhao, C. R., Ikka, T., Sawaki, Y., Kobayashi, Y., Suzuki, Y., Hibino, T., Sato, S., Sakurai, N., Shibata, D. & Koyama, H. (2009) Comparative transcriptomic characterization of aluminum, sodium chloride, cadmium and copper rhizotoxicities in Arabidopsis thaliana. *BMC Plant Biol*, 9, 32.
